# Supplementary material for: Maternal and/or direct supplementation with a combination of a casein hydrolysate and yeast β-glucan on post-weaning performance and intestinal health in the pig
Source: PLoS One. 2022 Jul 15;17(7):e0265051. doi: 10.1371/journal.pone.0265051 (PMC9286230; doi:10.1371/journal.pone.0265051)
Supplement: S1 File — (least-square mean values with their standard error of means). *A total of ten replicates were used per treatment. (PDF) [file pone.0265051.s001.pdf]

	Treatment*			
	Basal	Supplemented	SEM	P-value
**Phylum**				
Firmicutes	82.265	93.108	2.976	0.0188
Proteobacteria	11.344	1.687	0.724	<0.0001
Actinobacteria	1.027	1.740	0.373	0.1933
Spirochaetes	4.147	2.851	0.588	0.1463
Synergistetes	0.008	0.189	0.086	0.3752
Tenericutes	0.742	0.205	0.205	0.1321
**Family**				
Erysipelotrichaceae	4.375	3.601	0.632	0.403
Lachnospiraceae	14.057	18.725	1.286	0.019
Streptococcaceae	0.008	1.265	0.201	0.157
Peptococcaceae	0.031	0.234	0.107	0.288
Coriobacteriaceae	0.418	1.091	0.271	0.107
Peptostreptococcaceae	0.396	0.333	0.191	0.818
Lactobacillaceae	5.037	21.343	1.108	<0.001
Enterobacteriaceae	2.300	1.337	0.421	0.139
Clostridiales_Family_XIII__Incer	0.443	0.562	0.225	0.713
Micrococcaceae	0.088	0.054	0.084	0.786
Desulfovibrionaceae	0.058	0.108	0.091	0.705
Clostridiaceae	25.588	18.536	1.480	0.004
Eubacteriaceae	1.889	1.160	0.387	0.215
Hungateiclostridiaceae	1.049	0.792	0.303	0.563
Ruminococcaceae	10.279	11.473	1.048	0.430
Oscillospiraceae	2.270	2.544	0.493	0.698
Acidaminococcaceae	0.077	0.732	0.184	0.067
Veillonellaceae	0.050	0.249	0.117	0.301
Bifidobacteriaceae	0.592	0.864	0.271	0.487
Atopobiaceae	0.121	0.050	0.090	0.617
Synergistaceae	0.011	0.279	0.104	0.288
Carnobacteriaceae	0.161	0.076	0.106	0.604
Christensenellaceae	12.312	8.930	1.027	0.035
Spirochaetaceae	5.371	3.771	0.673	0.118
Anaeroplasmataceae	0.917	0.152	0.209	0.064
Planococcaceae	1.511	0.346	0.283	0.028
Moraxellaceae	9.072	0.552	0.578	<0.001
**Genus**				
Turicibacter	3.578	2.273	0.536	0.113
Coprococcus	3.380	4.204	0.619	0.357
Flavonifractor	2.430	1.537	0.442	0.184
Beduinibacterium	1.258	1.191	0.351	0.894
Streptococcus	0.008	1.340	0.206	0.152
Holdemanella	0.065	0.483	0.154	0.134

Peptococcus	0.031	0.235	0.107	0.287
Murimonas	0.174	0.212	0.140	0.849
Collinsella	0.429	1.150	0.277	0.093
Romboutsia	0.150	0.126	0.118	0.888
Lactobacillus	4.975	21.083	1.102	<0.001
Mediterraneibacter	2.929	3.069	0.550	0.860
Anaerovorax	0.021	0.048	0.058	0.751
Lachnoclostridium	0.127	0.578	0.180	0.129
Erysipelothrix	0.194	0.148	0.130	0.810
Intestinimonas	0.897	1.341	0.336	0.362
Falcatimonas	0.013	0.033	0.048	0.781
Clostridium	25.562	17.898	1.467	0.002
Eubacterium	1.901	1.128	0.385	0.189
Holdemania	0.572	0.562	0.239	0.976
Gemmiger	0.056	1.691	0.252	0.017
Ihubacter	0.418	0.434	0.207	0.956
Oscillibacter	2.282	2.450	0.489	0.811
Ruminococcus	1.505	1.593	0.395	0.876
Acetanaerobacterium	1.350	1.700	0.392	0.534
Megasphaera	0.025	0.034	0.055	0.909
Bifidobacterium	0.596	0.844	0.270	0.521
Salmonella	0.114	0.054	0.090	0.663
Kineothrix	3.526	1.424	0.482	0.012
Ruminiclostridium	1.034	0.556	0.278	0.263
Olsenella	0.124	0.050	0.090	0.603
Peptoclostridium	0.249	0.199	0.150	0.819
Anaerotignum	0.045	0.289	0.121	0.246
Christensenella	11.128	7.497	0.959	0.018
Niameybacter	0.111	1.765	0.272	0.009
Metabacterium	0.298	4.201	0.424	0.000
Pseudoflavonifractor	2.616	1.862	0.471	0.283
Cellulosilyticum	1.230	1.164	0.347	0.894
Treponema	5.444	3.698	0.672	0.091
Butyrivibrio	0.216	0.199	0.144	0.933
Anaerocolumna	0.558	0.163	0.180	0.197
Anaeroplasma	0.918	0.148	0.209	0.063
Sporobacter	0.858	0.665	0.276	0.632
Butyricicoccus	0.265	0.205	0.153	0.787
Oscillospira	0.819	0.998	0.303	0.681
Acinetobacter	9.431	0.547	0.586	<0.001
Eisenbergiella	0.355	0.127	0.149	0.348
Pseudobutyrivibrio	0.111	0.085	0.099	0.859
Flintibacter	0.055	0.015	0.056	0.676
Caryophanon	0.322	0.034	0.116	0.248

Enterocloster	0.119	0.022	0.077	0.492
