# Supplementary material for: Maternal and/or direct supplementation with a combination of a casein hydrolysate and yeast β-glucan on post-weaning performance and intestinal health in the pig
Source: PLoS One. 2022 Jul 15;17(7):e0265051. doi: 10.1371/journal.pone.0265051 (PMC9286230; doi:10.1371/journal.pone.0265051)
Supplement: S2 File — BB basal sow, basal pig; BS basal sow, supplemented pig; SB supplemented sow, basal pig; SS supplemented sow, supplemented pig; LT, lactation diet; PW, post-weaning diet. *A total of ten replicates were used per treatment. (PDF) [file pone.0265051.s002.pdf]

	Treatment*					P		
	BB	BS	SB	SS	SEM	Sow	Wean	Sow x Wean
**Phylum**								
Firmicutes	66.731	68.638	82.102	83.441	3.874	0.001	0.674	0.909
Bacteroidetes	18.091	21.347	11.461	12.584	1.767	0.001	0.281	0.760
TM7	0.007	0.014	0.048	0.063	0.076	0.638	0.893	0.956
Proteobacteria	11.944	5.982	3.519	1.802	1.020	<0.001	0.010	0.962
Spirochaetes	0.805	2.345	1.531	0.505	0.489	0.351	0.967	0.032
Actinobacteria	1.999	1.195	1.010	1.504	0.530	0.567	0.883	0.255
Fibrobacteres	0.016	0.096	0.012	0.007	0.070	0.708	0.882	0.767
Deferribacteres	0.224	0.037	0.210	0.041	0.148	0.993	0.339	0.962
**Family**								
Lachnospiraceae	11.269	13.340	16.995	13.160	1.650	0.124	0.727	0.102
Clostridiaceae	5.537	7.994	10.666	19.552	1.439	<0.001	0.005	0.438
Peptostreptococcacea	0.013	0.033	0.029	0.060	0.080	0.806	0.773	0.968
Mogibacteriaceae	0.255	0.510	0.393	0.180	0.254	0.719	0.957	0.391
Turicibacteriaceae	0.012	0.059	0.100	0.300	0.136	0.444	0.581	0.915
Erysipelotrichaceae	0.227	0.278	0.189	0.098	0.196	0.579	0.836	0.697
Streptococcaceae	0.013	0.025	0.009	0.015	0.055	0.908	0.888	0.989
Lactobacillaceae	28.310	16.725	31.313	23.917	2.225	0.024	0.001	0.181
Veillonellaceae	0.192	0.118	0.087	0.116	0.158	0.761	0.936	0.770
Peptococcaceae	0.060	0.032	0.049	0.020	0.088	0.894	0.762	0.957
Hellcobacteraceae	0.102	0.091	0.022	0.292	0.146	0.919	0.517	0.479
Campylobacteraceae	8.559	2.842	0.080	0.783	0.646	0.003	0.495	0.062
S24-7	3.368	3.321	3.732	2.257	0.793	0.588	0.327	0.354
BS11	0.006	0.497	0.012	0.024	0.117	0.779	0.516	0.633
p-2534-18B5	0.504	1.167	0.017	0.179	0.262	0.170	0.397	0.685
RF16	0.161	0.335	0.048	0.006	0.143	0.408	0.838	0.660
Porphyromondaceae	1.306	0.592	0.225	0.145	0.309	0.075	0.470	0.836
Bacteriodaceae	0.031	0.414	0.026	0.005	0.118	0.544	0.902	0.573
Prevotellaceae	7.673	5.289	2.567	6.586	1.033	0.051	0.188	0.006
Paraprevotellaceae	3.018	5.343	2.143	1.901	0.770	0.024	0.424	0.227
F16	0.009	0.017	0.058	0.072	0.082	0.607	0.896	0.949
Succinivibrionaceae	0.041	0.051	0.010	0.075	0.089	0.858	0.697	0.751
Pasteurellaceae	0.024	0.062	0.017	0.077	0.091	0.983	0.635	0.916
Enterbacteriaceae	2.173	1.164	0.121	0.131	0.365	0.015	0.774	0.709
Alcaligenaceae	0.020	0.008	0.001	0.002	0.035	0.812	0.980	0.934
Spirochaetaceae	0.925	3.022	1.840	0.575	0.538	0.275	0.981	0.015
Coriobacteriaceae	2.373	1.502	1.249	1.717	0.581	0.480	0.845	0.286
Fibrobacteraceae	0.020	0.110	0.015	0.008	0.076	0.687	0.883	0.745
Deferribacteraceae	0.244	0.048	0.293	0.045	0.164	0.974	0.288	0.936
Desulfovibrionaceae	0.115	0.322	0.153	0.397	0.215	0.813	0.346	0.972
Christensenellaceae	0.623	1.165	0.749	0.990	0.417	0.983	0.370	0.729

Ruminococcaceae	22.770	33.463	26.771	26.319	2.332	0.657	0.049	0.033
**Genus**								
Ruminococcus	0.061	0.465	0.393	0.360	0.241	0.473	0.387	0.347
Roseburia	1.239	1.151	1.562	1.378	0.515	0.606	0.802	0.948
Dorea	1.483	2.343	1.370	1.188	0.560	0.315	0.673	0.424
SMB53	0.023	0.149	0.071	0.098	0.125	0.860	0.579	0.697
Turicibacter	0.019	0.214	0.174	0.659	0.205	0.366	0.310	0.768
Eubacterium	0.074	0.108	0.145	0.049	0.135	0.968	0.826	0.651
p-75-a5	0.026	0.116	0.075	0.031	0.106	0.951	0.892	0.586
PSB-M-3	0.019	0.026	0.043	0.007	0.066	0.943	0.835	0.769
Bulleidia	0.052	0.193	0.092	0.096	0.143	0.966	0.661	0.680
Streptococcus	0.022	0.075	0.017	0.032	0.082	0.840	0.734	0.918
Lactobacillus	45.526	33.565	55.967	51.153	3.038	0.000	0.009	0.127
Phascolarctobacterium	0.092	0.061	0.134	0.108	0.139	0.753	0.837	0.950
Mitsuokella	0.166	0.062	0.022	0.069	0.119	0.642	0.970	0.607
Anaerovibrio	0.047	0.140	0.025	0.034	0.104	0.648	0.760	0.860
Peptococcus	0.101	0.072	0.096	0.044	0.123	0.874	0.743	0.896
Helicobacter	0.200	0.161	0.039	0.632	0.206	0.927	0.368	0.296
Compylobacter	12.409	5.383	0.152	1.532	0.835	0.0003	0.246	0.021
Parabacteroides	1.657	0.991	0.391	0.320	0.388	0.049	0.561	0.799
Bacteroides	0.046	0.733	0.074	0.012	0.162	0.470	0.853	0.364
YRC22	0.022	0.568	0.074	0.004	0.138	0.640	0.968	0.446
Prevotella-1	12.372	10.641	5.921	14.433	1.455	0.152	0.020	0.002
Prevotella-2	3.649	8.394	2.541	3.299	0.919	0.014	0.032	0.238
CF231	0.994	0.783	1.640	0.604	0.440	0.804	0.213	0.437
Succinivibrio	0.059	0.103	0.021	0.158	0.124	0.883	0.529	0.721
Actinobacillus	0.040	0.119	0.037	0.161	0.127	0.950	0.495	0.919
Sutterella	0.030	0.014	0.002	0.004	0.045	0.766	0.992	0.916
Treponema	1.507	5.829	3.653	1.236	0.745	0.299	0.669	0.001
Slackia	0.100	0.162	0.116	0.101	0.154	0.901	0.898	0.817
Collinsella	1.023	1.169	0.846	1.299	0.464	0.924	0.528	0.739
Fibrobacter	0.035	0.207	0.035	0.018	0.108	0.621	0.821	0.622
Mucispirillum	0.347	0.095	0.564	0.093	0.218	0.841	0.192	0.827
Desulfovibrio	0.179	0.577	0.306	0.801	0.294	0.583	0.185	0.894
Clostridium	0.767	1.655	0.613	0.308	0.391	0.122	0.945	0.229
Ruminococcus	6.015	8.283	5.494	3.799	1.076	0.037	0.900	0.091
Faecalibacterium	0.126	0.651	0.081	0.621	0.250	0.825	0.108	0.858
Oscillospira	2.250	3.332	4.556	2.244	0.778	0.567	0.561	0.055
Lachnospira	0.682	2.398	3.277	2.083	0.629	0.068	0.287	0.032
Coprecoccus	1.503	4.753	3.419	3.801	0.806	0.280	0.032	0.068
